# A Laboratory Investigation of the Suspension, Transport, and Settling of Silver Carp Eggs Using Synthetic Surrogates

**DOI:** 10.1371/journal.pone.0145775

**Published:** 2015-12-29

**Authors:** Tatiana Garcia, Carlo Zuniga Zamalloa, P. Ryan Jackson, Elizabeth A. Murphy, Marcelo H. Garcia

**Affiliations:** 1 U.S. Geological Survey, Illinois Water Science Center, Urbana, IL, 61801, United States of America; 2 Dept. of Civil and Environmental Engineering, University of Illinois at Urbana-Champaign, Urbana, IL, 61801, United States of America; University of Washington, UNITED STATES

## Abstract

Asian carp eggs are semi-buoyant and must remain suspended in the water to survive, supported by the turbulence of the flow, until they hatch and develop the ability to swim. Analysis of the transport and dispersal patterns of Asian carp eggs will facilitate the development and implementation of control strategies to target the early life stages. Experimenting with Asian carp eggs is complicated due to practical issues of obtaining eggs in close proximity to experimental facilities and extensive handling of eggs tends to damage them. Herein, we describe laboratory experiments using styrene beads (4.85 mm diameter) as synthetic surrogate eggs to mimic the physical properties of water-hardened silver carp eggs. The first set of experiments was completed in a rectangular vertical column filled with salt water. The salinity of the water was adjusted in an iterative fashion to obtain a close approximation of the fall velocity of the styrene beads to the mean fall velocity of silver carp water-hardened eggs. The terminal fall velocity of synthetic eggs was measured using an image processing method. The second set of experiments was performed in a temperature-controlled recirculatory flume with a sediment bed. The flume was filled with salt water, and synthetic eggs were allowed to drift under different flow conditions. Drifting behavior, suspension conditions, and settling characteristics of synthetic eggs were observed. At high velocities, eggs were suspended and distributed through the water column. Eggs that touched the sediment bed were re-entrained by the flow. Eggs saltated when they touched the bed, especially at moderate velocities and with a relatively flat bed. At lower velocities, some settling of the eggs was observed. With lower velocities and a flat bed, eggs were trapped near the walls of the flume. When bedforms were present, eggs were trapped in the lee of the bedforms in addition to being trapped near the flume walls. Results of this research study provide insights about transport, suspension, and dispersion of silver carp eggs. The knowledge gained from this study is useful to characterize the critical hydrodynamic conditions of the flow at which surrogates for silver carp water-hardened eggs settle out of suspension, and provides insight into how eggs may interact with riverbed sediments and morphology.

## Introduction

In recent years, the population of Asian carp has increased exponentially in the Mississippi River Basin [1–4]. The population will continue to increase because Asian carp can lay thousands of eggs and have the ability to spawn up to three times per year [5]. This population increase is a concern because of the identification of potential connections between the Mississippi River Basin and the Great Lakes Basin, which does not currently have known populations of silver and bighead carp [6]. If Asian carp move into the Great Lakes Basin, they could cause negative ecological and economic impacts [7]. An increased understanding of Asian carp spawning and egg transport will lead to new approaches for identifying rivers suitable for Asian carp reproduction, and designing control methods.

Should Asian carp invade the Great Lakes, they will require rivers to spawn. Drifting silver (*Hypophthalmichthys molitrix*) and bighead carp (*Aristichyths nobilis*) eggs are semibuoyant, and need to be in suspension to hatch [4, 8–10]. This fact has led researchers to identify potential spawning rivers through progressively more detailed analyses. Kolar et al. [4] used a coarse filter to identify 22 Great Lakes tributaries as potential spawning rivers. This assessment was based on the criterion that states a potential spawning river must be at least 100 *km* long for successful recruitment. More recently, Kocovsky et al. [11] evaluated the spawning potential of several Lake Erie tributaries. The authors studied temperatures and velocity time series of selected rivers based on the identification of flood events considered favorable to Asian carp spawning. They defined temperature suitable for spawning as greater than 21°C, and defined flood events favorable for spawning when the maximum velocity exceeded 0.7 m/s. The authors concluded that the largest western and central Lake Erie tributaries are hydrologically and thermally suitable for Asian carp reproduction. Murphy and Jackson [12] used field measurements of velocity and temperature for four Great Lakes tributaries and found that river reaches as short as 25 km could permit suspension until eggs hatch, and velocities as low as 0.15 to 0.25 m/s could maintain the eggs in suspension. Garcia et al. [13, 14] substantiated these conclusions using the FluEgg drift model and shortly thereafter, Chapman et al. [15] validated these conclusions with the first evidence of grass carp reproduction in the Great Lakes.

Important factors believed to affect the spawning and recruitment of Asian carp include the following: water level fluctuation, turbulence, velocity, temperature, and turbidity [4, 16]. Asian carp spawning grounds are in areas characterized by turbulent currents and mixing waters like river confluences, rock rapids, behind sandbars, stonebeds or islands, and downstream of dams and spillways where bubbles and eddies are present [4, 9, 16, 17]. Spawning grounds of Asian carp are generally characterized by turbid flowing water (0.25 to 3.0^m^/_s_) [4, 10, 16, 18]. Temperatures at spawning grounds reportedly range between 18 and 30°C [4, 9, 10, 16].

Following fertilization, Asian carp eggs begin to absorb water, thereby increasing in size and decreasing their density over time [1, 16, 18–20]. The diameter increases and the fall velocity decreases with time until an asymptotic value is reached. Four hours after fertilization, the egg diameter remains relatively constant [1, 21]. Yi et al. (1988) (English translation available in Chapman [8]) collected and analyzed Asian carp (grass, black, silver and bighead carps) eggs and larvae from the Yangtze River in the 1960s. From this study, the authors found that silver carp eggs were the smallest, and bighead carp eggs were the largest, compared with other species. The diameter of silver carp eggs ranged between 3.5 and 6.4 mm, with a mean value ($\bar{D}$) of 5.05 mm and a standard deviation (*σ*) of 0.52 mm. Bighead carp egg diameters varied from 4.9 to 6.7 mm ($\bar{D}=5.82\text{mm};\sigma =0.42\text{mm}$). Chapman and George [1, 21] cultured silver and bighead carp under regulated conditions. Important biological factors such as size, diameter, growth rate, and fall velocities were documented. Before fertilization, silver and bighead carp egg diameters were 1.4 and 1.6 mm, respectively. After fertilization, silver carp egg diameters ranged between 3.2 and 5.4 mm ($\bar{D}=4.3\text{mm};\sigma =0.45\text{mm}$). Bighead carp egg diameters varied from 3.6 to 6.7 mm ($\bar{D}=5.3\text{mm};\sigma =0.58\text{mm}$). Lenaerts et al. [22] collected and analyzed Asian carp eggs from the Wabash River in Indiana in 2012 and 2013, and found the average silver carp egg diameter over both years was 3.1 mm, with the eggs from 2012 generally smaller than those collected in 2013. Bighead and hybrid carp both had eggs that were larger on average than the silver carp eggs, but had average diameters less than 3.5 mm, over the two sampled years.

Previous studies [4, 11] assessed Asian carp reproduction using estimates of the critical velocity to maintain eggs in suspension based on observations of known spawning grounds, rather than physically based studies. Studies with Asian carp eggs are difficult to perform, and require culturing eggs in a laboratory or collecting eggs from the field, and transporting them to the lab without delay or damage. The difficulties of working with live eggs have led experimentalists to seek egg surrogates for laboratory experiments. Dudley and Platania [23] experimented with different potential materials to mimic the physical properties (specific gravity and settling velocity) of drifting semibuoyant Cyprinidae eggs. The Cyprinidae eggs had a diameter of 2.672 mm, with specific gravity equal to 1.00589, and a terminal fall velocity of 9.291 mm/s. As a result of these experiments, the authors concluded that semibuoyant nylon 12 particles (specific gravity = 1.005) were the optimum material to mimic the fish eggs.

Following the methods of Dudley and Platania [23], synthetic eggs can be used in the study of the fate and transport of Asian carp eggs. Synthetic eggs can be used to identify the critical hydrodynamic conditions at which the eggs, for different developmental stages, remain in suspension. Throughout their pre-hatching development, silver carp eggs have a lower settling velocity than bighead carp eggs [21]. Therefore, they require lower velocities and turbulence to remain in suspension. We selected silver carp as the critical species to evaluate. Assuming Asian carp select highly turbulent areas to spawn to maintain their non-water-hardened eggs in suspension, the critical developmental stage begins when the eggs become water-hardened. Laboratory experiments with synthetic eggs were performed in the Ven Te Chow Hydrosystems Laboratory, University of Illinois at Urbana-Champaign.

The purposes of this study were: i) to identify a material that can mimic the physical characteristics of silver carp eggs, ii) to design and carry-out flume experiments using synthetic eggs to better understand egg transport under turbulent flow conditions, which are similar to preferred spawning conditions in streams, iii) to characterize, qualitatively, both egg-bed interaction and egg burial processes, and iv) to evaluate suspension and settling dynamics of water-hardened eggs under different flow velocities.

## Materials and Methods

Commercially available styrene spheres were used as synthetic surrogate eggs. The spheres had a diameter of 4.85mm, a roundness tolerance of approximately ±0.015 mm and a density approximately equal to 1040.9^kg^/_m^3^_. Synthetic eggs were purchased from Engineering Laboratories, Inc. (www.plasticballs.com). Because the styrene spheres were not precision grade, a thorough characterization of the density and diameter was required. The diameter of synthetic eggs was determined by taking a picture of a random sample of 36 particles, which were placed over a white background next to a rectangular grid. The picture frame was analyzed using custom *MATLAB* scripts (Mathworks, Natick, MA, USA). This process produced a measure of the mean diameter.

To compensate for the high specific gravity of the styrene spheres relative to water-hardened silver carp eggs, the density of the water was modified using salt. Increasing the density of the water using salt increases the buoyancy of the spheres and reduces the settling velocity. The density of the water was measured using an Anton Paar DMA 500 density meter.

### Experiments with synthetic surrogate eggs in stagnant water

Results from this experiment provided the necessary water density and salinity concentration to be used in laboratory experiments with moving water using the chosen synthetic eggs.

A technique to calculate the terminal fall velocity of the synthetic eggs was developed. Terminal fall velocity was estimated in a rectangular 1.6m tall, 0.2m long, and 0.1m wide clear acrylic settling column. The column was filled with salt water. The salinity of the water was adjusted in an iterative process such that the settling velocity of the synthetic eggs would mimic the settling velocity of water-hardened silver carp eggs as determined in the laboratory experiments of Chapman and George [21]. Temperature was measured at the beginning and end of each experiment. Prior to the experiment, the water was well mixed to avoid stratification caused by salinity or thermal differences. A funnel was located in the middle of the section at the top of the column. Individual synthetic eggs were passed through the funnel and were tracked as they fell through the settling column. The terminal fall velocity of individual synthetic eggs was calculated using a particle tracking system.

**Fig 1:**
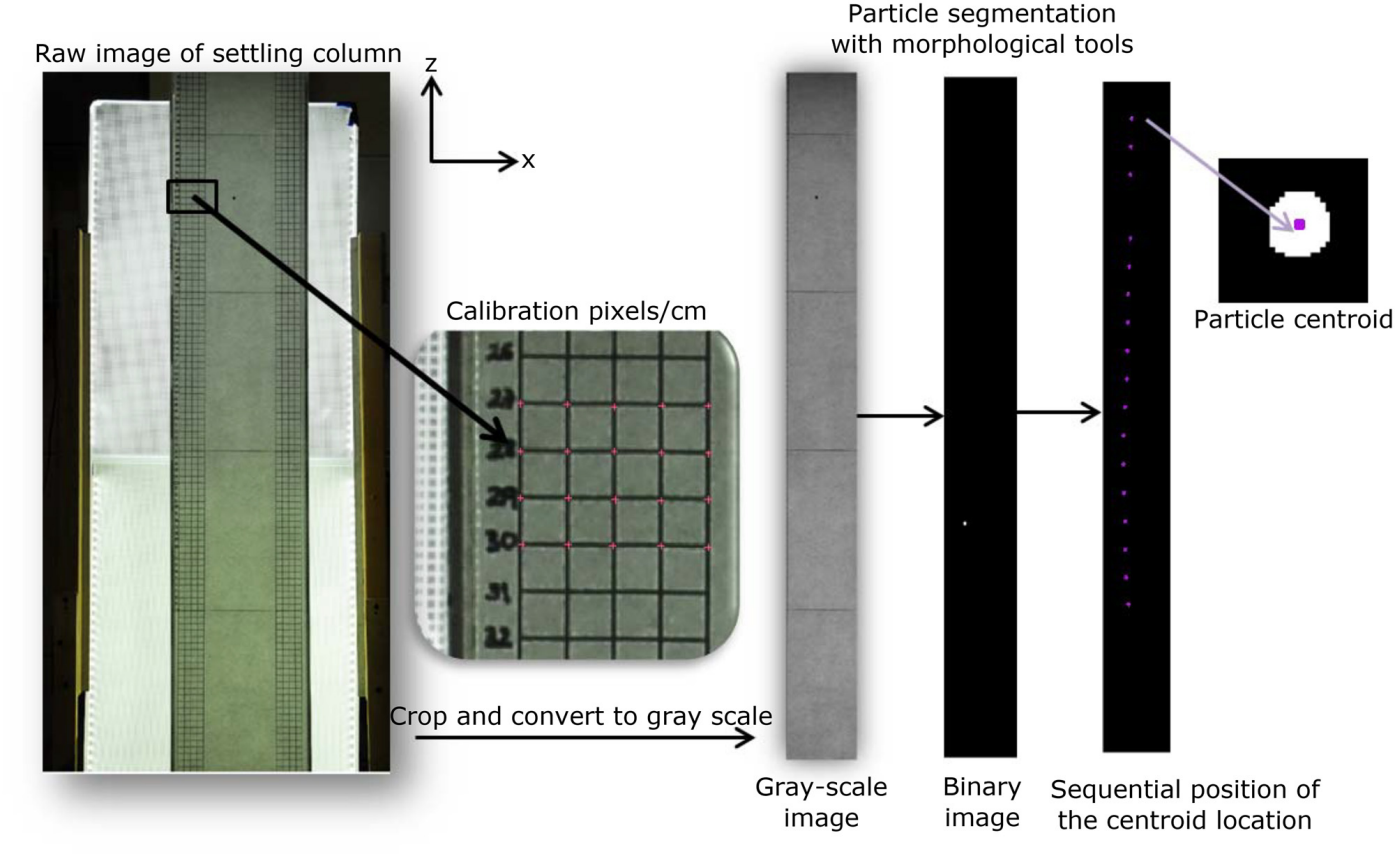
Steps in the particle image tracking code.

A 1 cm x 1 cm vertical grid was located in the right and left edges of the column for later calibration (Fig 1). The settling column was illuminated with a system of two 46 × 61-cm LED Edge Lit Panels (purchased from Knema, LLC (http://knema.com)). The LED panels were located behind the settling column to produce a uniform illumination field. The settling column was rectangular to minimize horizontal visual distortion.

Consecutive high-resolution images of each particle were taken at 9 frames per second with a Nikon D200 camera. The images were taken with a focal length of 35mm, exposure time of 1/160 seconds and an aperture of f/8 capturing the particles at different times as they fell through the water column. The sequence of images was used to track the path of individual particles. The location (x, z) of the particles and the time interval between images were used to calculate the terminal fall velocity at different depths of the column. The terminal fall velocity was computed after initial acceleration (approximately 0.5m below water surface). Fifty realizations were completed to develop a representative sample of the fall velocity.

Custom particle image processing *MATLAB* routines were developed to track the position of individual particles in a series of consecutive picture frames. The methodology used is based on visual detection of particles in each frame. The time at which each image was captured was obtained from the camera date database. The custom *MATLAB* routines convert images first to gray scale and then to black and white (binary images). The binary images are processed using morphological processing tools to identify the centroid of each particle. The centroid of the particle is in pixel units; therefore, a calibration is needed to obtain the location of the particles in metric units (see Fig 1).

Initially, a long ruler was placed inside the center of the settling column for the purpose of camera calibration. A ratio of pixels/cm was calculated for various segments of the ruler and for the grid located in the back of the column. The error found between the different pixel/cm ratio measured in the center and the back of the column was less than 3 pixels/cm, which is well within the uncertainty of this methodology. From these measurements, an average calibration scale between pixels and meters was calculated. The particle image tracking code calculates the fall velocity per pair of two sequential images, as follows:

$$\begin{array}{c} V_{s}=\frac{Z^{n+1}-Z^{n}}{t^{n+1}-t^{n}} \end{array}$$ (1)

where, *V*_*s*_ is the terminal fall velocity [^m^/_s_], *Z* is the vertical location of the particle centroid (depth from water surface to particle location) [m], *t* is the time at which the picture frame was captured [*seconds*], and *n* represents the time step [*unitless*].

The particle image tracking code returned information on fall velocity as a function of depth. Later, the mean terminal fall velocity along the settling column depth was calculated. The minimum number of particles needed to estimate a statistically significant mean terminal fall velocity of the synthetic surrogate eggs was determined. Settling column trials (realizations) were done with an additional measurement of particle settling velocity being tested in each subsequent trial until the mean terminal fall velocity reached an asymptotic value. The terminal fall velocities obtained from the particle image tracking code were compared with four equations found in literature to calculate terminal fall velocities in quiescent water (a summary can be found in Garcia [24]): (i) Iteration Method, (ii) Dietrich, (iii) Jimenez and Madsen, and (iv) Soulsby.

### Experiments with synthetic surrogate eggs in flowing water

**Fig 2:**
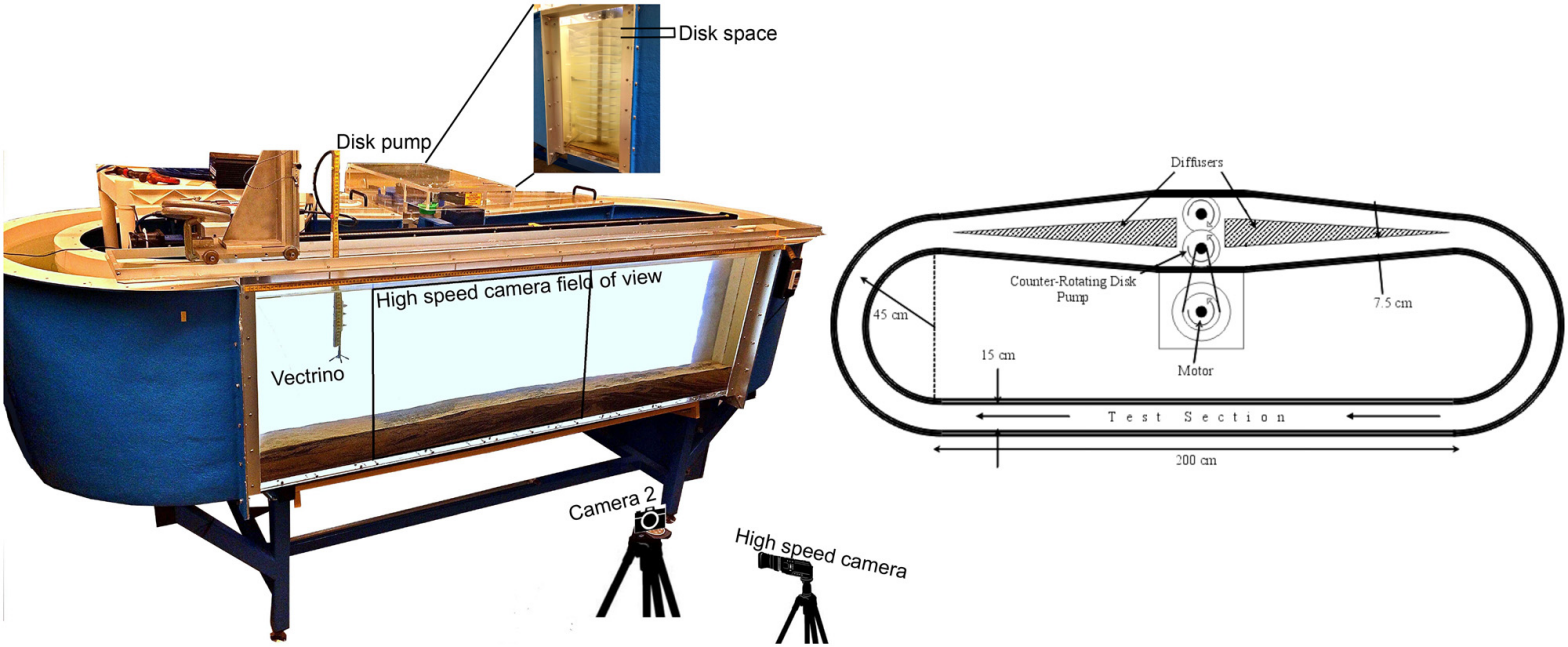
Odell-Kovasznay flume—Ven Te Chow Hydrosystems Laboratory.

Experiments with synthetic surrogate eggs in flowing water were performed in an Odell-Kovasznay style flume [25], a temperature-controlled recirculatory flume built originally for stratified flow experiments at the University of Illinois [26]. The flow in the flume is controlled by a disk pump, which runs at different rotation rates resulting in different ranges of flow velocities. Turbulence is produced by the disk pump, and shear is generated by the walls of the flume and the sediment bed. The dimensions of the Odell-Kovasznay flume are shown in Fig 2. The transparent test section is 2 m long, 0.15 m wide, and 0.6 m deep. The disk pump, also shown in Fig 2, has disks uniformly distributed through the depth of the flume with a disk spacing of 3.4 cm. The pump disks were separated carefully such that there was enough surface area to generate a wide range of flow velocity in the flume, yet, disk spacing ensures no damage to the synthetic eggs.

The flume was filled with salt water to adjust the specific gravity of synthetic eggs such that the physical properties of water-hardened silver carp eggs were matched. A mixture of walnut-shells and sand was used as a sediment bed in the Odell-Kovasznay flume. The sediment bed artificially reproduced deposition and entrainment of eggs, and reproduced egg-sediment interaction that likely occurs when eggs settle to the bottom of a river. In addition, the sediment substrate produced shear, generated by the roughness of the sediment bed. This shear mimics some of the hydrodynamic effects present in natural rivers.

Measurements of the water velocity were performed using a Nortek Vectrino acoustic Doppler velocimeter with a Velmex positioner, capable of measuring three-component velocity (*u*, *v*, *w*) at precise positions in the water column [27]. The sampling volume of the Vectrino has a diameter of 6.0mm, and a variable height of 3 − 15mm. The Vectrino was setup with a transmit length of 1.8mm.

The experiments performed in the Odell-Kovasznay flume included: (i) measurement of the settling velocity of synthetic eggs in stagnant water, (ii) qualitative observation of the transport of synthetic eggs at different flow velocities, (iii) estimation of the vertical distribution of synthetic eggs drifting at a given velocity, and (iv) tracking of synthetic eggs and egg trajectory at a given velocity.

Initially, synthetic eggs were released in the flume with stagnant water to ensure the experimental setup was reproducing the conditions determined from the column experiments required to imitate the settling velocity of water-hardened silver carp eggs. Although the flume was not deep enough to reach terminal fall velocity, it gave a good estimate of the settling velocity. Eggs were released through a funnel in the middle of the test section. As the eggs settled, the locations of synthetic eggs at different time steps were captured by picture frames with a high-speed camera. The picture frames were analyzed with the *MATLAB* particle tracking toolbox, PTVlab [28].

The synthetic eggs were then allowed to drift in the current under different velocity fields. The transport and dispersion of the eggs were analyzed qualitatively (documented by photographs and videos), and quantitatively. The quantitative analysis was performed by capturing the location of synthetic eggs with a high-speed camera. A given egg location was tracked through a series of picture frames using the PTVlab tracking toolbox.

Egg transport, along with egg suspension and settling dynamics, were observed and documented in pictures and videos; Fig 2 shows the position of camera 2 relative to the flume. Egg transport was analyzed under two conditions: relatively flat bed and a mobile bed with bedforms. Eggs were allowed to drift until a relatively steady state distribution of eggs was reached for each experiment. When the flow rate in the flume was changed, the flume was allowed to run for about 10 minutes until the flow reached steady state. The velocity profile generated at different flow rates was measured with the Vectrino at the end of the high speed camera view area (see Fig 2). The Vectrino was placed far enough from the bend entering the test section to minimize the effect of secondary currents on velocity measurements. Shear velocity (u_*_, indicator of turbulence) estimates were calculated as $u_{*}=\sqrt{\frac{\tau_{b}}{\rho }}$ for further analysis. Estimates of bed shear stress (*τ*_*b*_) were calculated using the turbulent kinetic energy (TKE) method. The mean bed shear stress was estimated from the three-component turbulent velocity fluctuations (*u*′, *v*′, *w*′) as follows [29, 30]:

$$\begin{array}{c} \tau_{b}=C_{1}\rho \left[0.5\left(\bar{u^{\prime }{}^{2}}+\bar{v^{\prime }{}^{2}}+\bar{w^{\prime }{}^{2}}\right)\right] \end{array}$$ (2)

where *τ*_*b*_ is the mean bed shear stress [*Nm* ^2^], *ρ* is the water density [^kg^/_m^3^_], *u*′, *v*′ and *w*′ are the velocity fluctuations in the longitudinal, lateral and vertical directions, respectively [^m^/_s_], and *C*_1_ = 0.19 is a proportionality constant [unitless].

Detailed experiments using the high-speed camera were performed for a selected flow condition at which synthetic eggs were both in suspension and settled on the bed, suggesting it is a condition approaching the critical suspension threshold for the eggs. To estimate the vertical distribution of synthetic eggs, independent picture frames were taken every minute extending over 70 minutes. The centroid of every particle was identified in every picture frame using the PTVlab toolbox. The locations of the centroid of 2,405 independent particles were identified, and the vertical distribution of the eggs was derived from this information.

Finally, a set of 840 correlated pictures at a rate of 8 frames per second were taken, while the flume was operated at the selected representative flow condition. Images were processed using the PTVlab toolbox and trajectories from individual synthetic eggs were identified. The egg trajectory slopes were analyzed to study suspension and settling patterns as a function of the mean vertical location of the eggs.

## Results and Discussion

This section describes the results of the experiments using synthetic eggs first in stagnant water in the settling column, and then in the Odell-Kovasznay recirculatory flume. The synthetic eggs were selected to imitate water-hardened silver carp eggs physical characteristics corresponding to data on cultured silver carp from Chapman and George [21]. At a reference temperature of 22°C, the average mean ± one standard deviation diameter, specific gravity and fall velocity of water-hardened silver carp eggs, during this developmental stage (4 hours to 23 hours post-fertilization) are 4.7±0.35mm, 1.0017±0.0003 and 0.007±0.0006^m^/_s_, respectively [21].

### Terminal fall velocity of synthetic surrogate eggs in stagnant water

**Fig 3:**
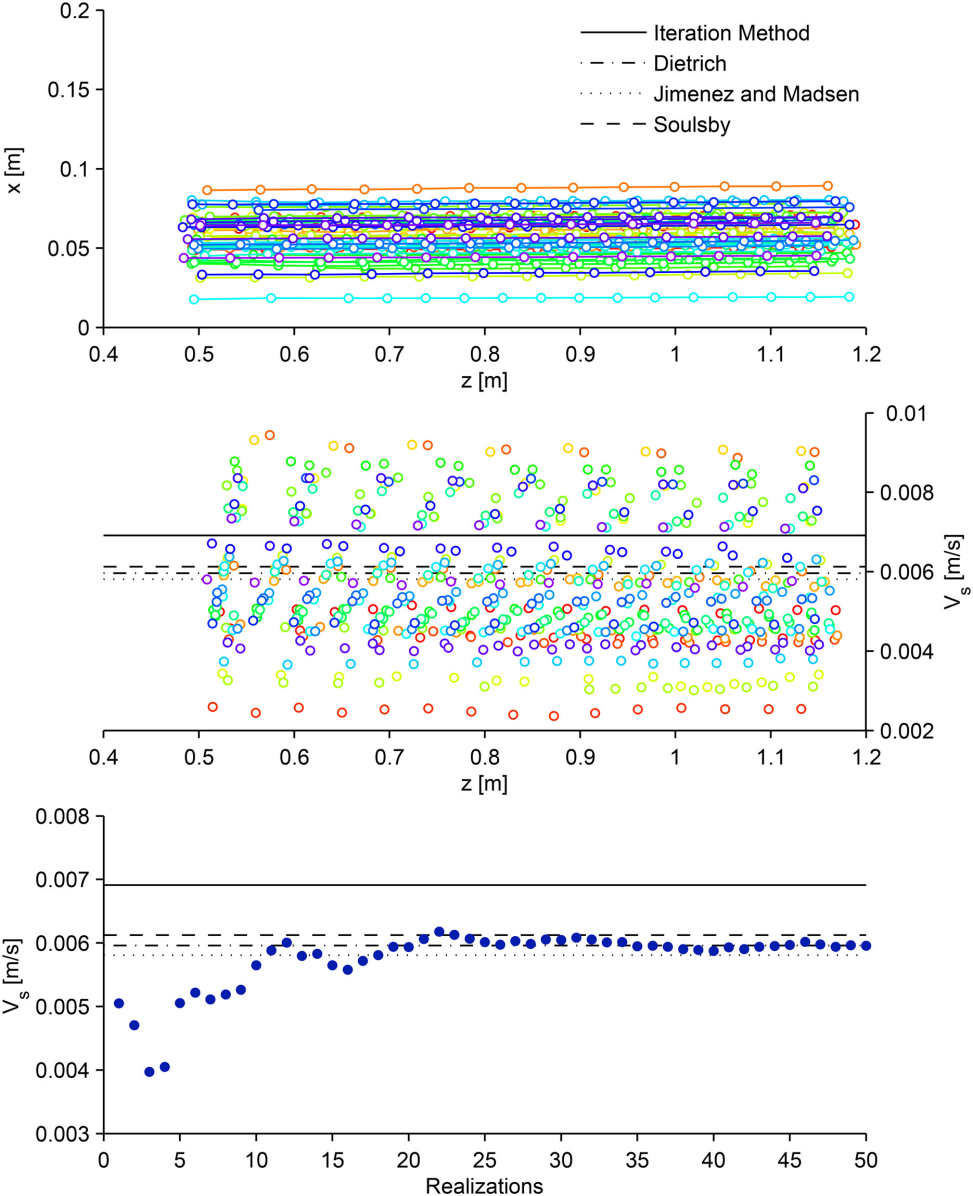
Synthetic surrogate water-hardened silver carp eggs fall velocity measurements. Settling paths for 50 synthetic eggs (particles) with 4.85 mm diameter and water specific gravity of 1.042 (different colors correspond to different synthetic eggs, circles represent egg location captured by photographs, connecting lines represent egg paths) (A); fall velocity of 50 synthetic eggs (different colors correspond to different synthetic eggs) (B); incremental mean terminal fall velocity of synthetic eggs (C).

Styrene particles in salt water with a mean diameter of 4.85mm (representative of water-hardened silver carp eggs) and a density and specific gravity of about 1040.9^kg^/_m^3^_ and 1.043, respectively, were found to have a similar fall velocity to water-hardened silver carp eggs. The salinity of the salt water was equal to 55.5 ppt with specific gravity of 1.042 and temperature of 22.8°C. We determined, experimentally, that at 50 cm below the water surface, the particles reached the terminal fall velocity (relatively constant speed). The terminal fall velocity of 50 synthetic eggs was analyzed as they settled through the column. Although all the eggs were placed in the column through a funnel (resulting in identical release points), every particle pursued a different path due to turbulence generated by the drag forces as the particles settled through the column (Fig 3A). Fig 3B illustrates fall velocity as a function of depth of the column for 50 synthetic eggs. Statistically, a sample of at least 25 synthetic eggs is required to reach an asymptotic mean value of the terminal fall velocity (Fig 3C). Results illustrate the mean terminal settling velocity of the synthetic eggs in the salt water solution (based on 50 realizations) is equal to 0.006±0.0016^m^/_s_. The terminal fall velocity results from the laboratory experiments are best represented with the Dietrich equation. This value of the mean terminal settling velocity indicates the synthetic eggs in salt water with a specific gravity of around 1.042 can mimic water-hardened silver carp eggs. By adjusting the salinity of the water, synthetic particles can be used to mimic eggs from other species at different developmental stages, provided the particles can be found to match the required egg diameter and the salinity adjustment is within the solubility limit of salt in water.

### Settling velocity of synthetic surrogate eggs in the Odell-Kovaznay flume in stagnant water

**Fig 4:**
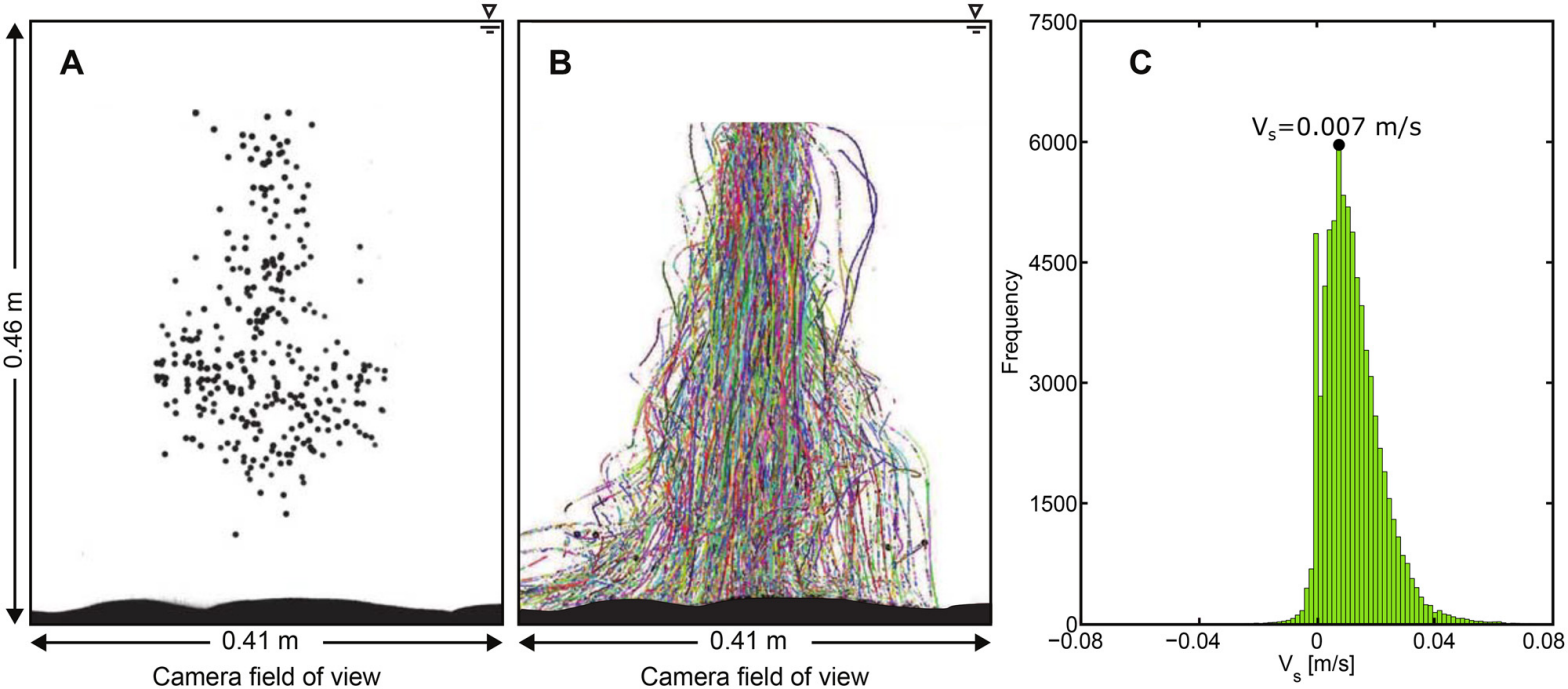
Experiments of settling velocity of synthetic eggs in the Odell-Kovasznay flume with stagnant water conditions. Photograph of the synthetic eggs settling in the Odell-Kovasznay flume (A). Paths of individual synthetic eggs (different colors correspond to different synthetic eggs) (B). Distribution of settling velocity (*V*_*s*_) of synthetic eggs [^m^/_s_] (C). Positive values indicate downward movement of particles.

The Odell-Kovasznay flume was filled with salt water with a specific gravity equal to 1.042, and a mean water depth and temperature equal to 0.46 m and 23.6°C, respectively. Approximately 350 synthetic eggs were released from the top of the flume in the middle of the field of view of the high speed camera (Fig 2). For this experiment, the water was stagnant to ensure the experimental set-up was reproducing roughly the same conditions as the vertical column. Fig 4A and 4B illustrate the turbulent diffusion due to drag forces generated as the synthetic eggs are settling. Fig 4B illustrates the paths of synthetic eggs as they sink in the flume; notice every egg takes a different path. The peak value of the settling velocity distribution of the synthetic eggs corresponds to a value of 0.007^m^/_s_ (Fig 4C), which is in agreement with water-hardened silver carp eggs settling velocities. The settling velocities measured in this experiment do not represent the terminal fall velocity of the eggs; however, they are a good indication of the ability of the synthetic eggs to mimic water-hardened silver carp eggs. A few synthetic eggs experienced upward movement (negative settling velocities). This upward movement occurred because small bubbles were attached to the surface of the synthetic eggs forcing them to briefly move upwards. However, by the end of the experiments all the particles settled to the bed.

### Qualitative observation of drifting synthetic surrogate eggs in the Odell-Kovaznay flume in moving water

**Fig 5:**
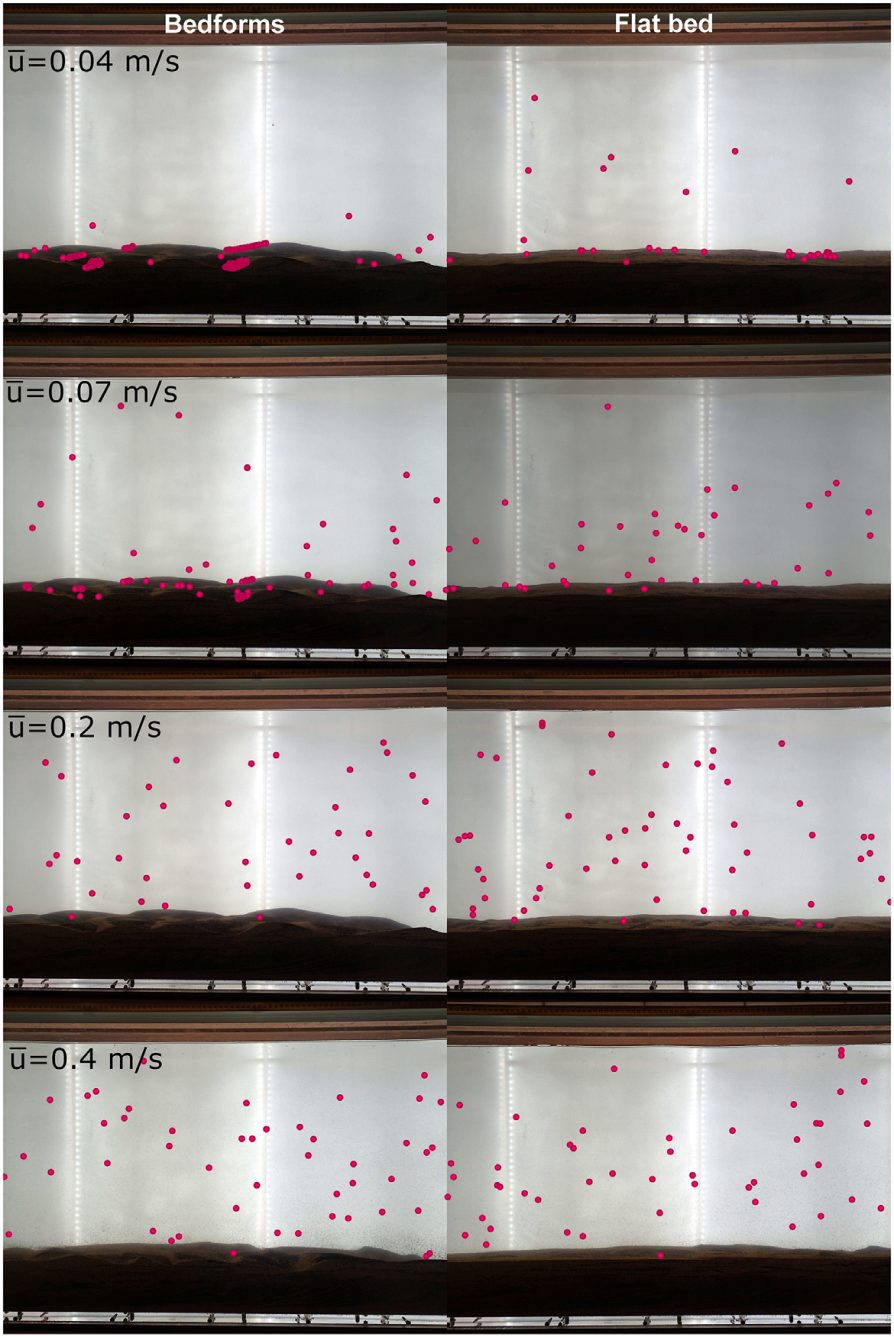
Mosaic of pictures taken at a given instant of time for two cases: bed with bedforms (left) and relatively flat bed (right). Every row represents a different velocity case: 0.04^m^/_s_ (u* = 0.002^m^/_s_), 0.07^m^/_s_ (u* = 0.004^m^/_s_), 0.2^m^/_s_ (u* = 0.008^m^/_s_), and 0.4^m^/_s_ (u* = 0.016^m^/_s_). Eggs size and color were enhanced for visualization.

Velocity distributions were measured in the Odell-Kovasznay flume using the Nortek Vectrino acoustic Doppler velocimeter. The flume was run for 10 minutes before every experiment to ensure the flow and the dispersion of the eggs reached steady state. The measured depth-averaged velocities in the flume for the four experiments were 0.04^m^/_s_, 0.07^m^/_s_, 0.2^m^/_s_, and 0.4^m^/_s_. The estimates of shear velocity calculated using the TKE method corresponding to the four flow conditions with a relatively flat bed were 0.002^m^/_s_, 0.004^m^/_s_, 0.008^m^/_s_, and 0.016^m^/_s_, respectively. Two cases were analyzed; one with a relatively flat bed and the other with bedforms. Fig 5 illustrates egg distribution for an instant of time. Video of synthetic eggs drifting under the different combinations of velocity and bed configuration are provided as supplementary material (S1 Video).

In the presence of bedforms and at a mean velocity equal to 0.04^m^/_s_ (u* = 0.002^m^/_s_), there were few eggs in suspension, and a large fraction of the eggs were trapped in the lee of the dunes; especially in areas near the walls of the flume. These areas are characterized by very low velocities. In the lee of the dunes, flow separation can create recirculation and dead zones, and shear near the flume walls causes very low, near-zero velocities. Therefore, it is very difficult for the trapped eggs to get re-entrained to the flow. However, at the same flow velocity and relatively flat bed conditions, fewer eggs were trapped on the bed compared to the case with bedforms. There were some instances where eggs were trapped due to imperfections of the bed (note: flat bed conditions still used a sediment bed and therefore, imperfections were present). In general, eggs that touched the bed demonstrated saltation processes where eggs rolled over the bed. However, it appears that the mixing near the bed was not strong enough to re-entrain settled eggs and transport them back into the upper part of the water column.

For the case in which bedforms were present, and a mean velocity equal to 0.07^m^/_s_ (u* = 0.004^m^/_s_), there were fewer eggs trapped along the bed compared to the case where velocities were equal to 0.04^m^/_s_ (u* = 0.002^m^/_s_). Also, the percentage of eggs in suspension was higher than in the previous case because there was more mixing to maintain the eggs in suspension, resulting in fewer eggs trapped on the bed. Eggs that settle and touch the bed bounced back into the water column, getting re-entrained into the flow relatively quickly. However, if an egg was trapped on the bed for an extended period of time, it was very difficult to get re-entrained into the flow. Under the same velocities, but with a relatively flat bed with no bedforms, eggs did not settle on the bottom. Instead, eggs saltated over the bed and never remained trapped in one place on the bed. In this case, there were more eggs in suspension than in the previous case (flat bed and 0.04^m^/_s_).

At mean velocities equal to 0.2^m^/_s_ (u* = 0.008^m^/_s_) and 0.4^m^/_s_ (u* = 0.016^m^/_s_), there were no noticeable differences between the cases with bedforms and the cases with a relatively flat bed. Almost all the eggs were in suspension. Occasionally, there were eggs near the bed; however; eggs were re-entrained rapidly to the flow. At velocities equal to 0.4^m^/_s_ (u* = 0.016^m^/_s_), the eggs simply moved faster and were re-entrained faster if they touched the bed.

### Estimation of the vertical distribution of synthetic surrogate eggs

**Fig 6:**
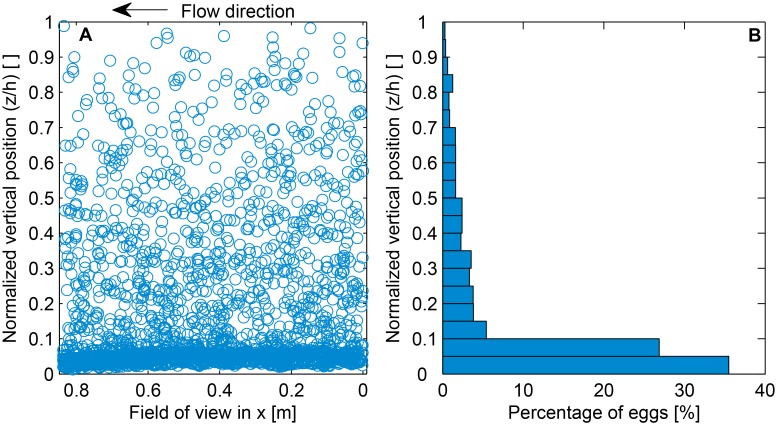
Location of the centroid of synthetic surrogate eggs obtained from independent pictures taken at 1 frame per minute (A). In (A) white space in the bottom $\left(\frac{z}{h}=0\right)$ is due to irregularities in the bed. Y-axis corresponds to the normalized vertical location of the eggs. Vertical distribution of the eggs (B). This figure is for the case at which the mean streamwise velocity was equal to 0.07^m^/_s_ (u* = 0.004^m^/_s_).

We selected the case of mean velocity equal to 0.07^m^/_s_ (u* = 0.004^m^/_s_) with a flat bed to explore drift trajectories and suspension and settling processes in more detail. Based on our observations, this is a representative case where both eggs in suspension and eggs near the bed can be studied, and it represents a critical hydrodynamic condition at or near the threshold for egg suspension. Independent images were taken every minute to avoid taking pictures of the same group of eggs. The location of the centroid of every egg in each picture was identified using the PTVlab toolbox (Fig 6A).

When the bed was relatively flat and at velocities as low as 0.07^m^/_s_ (u* = 0.004^m^/_s_), about 65% of the eggs remained in suspension, corresponding to a vertical centroid of the mass of eggs located at the lower 18% of the water column. (Fig 6). Previously, it was believed that velocities ranging from 0.25 to 0.9^m^/_s_, were required to maintain eggs in suspension [4, 10, 16, 18]. Kocovsky et al. [11] used a velocity threshold of 0.[7]^m^/_s_ required to maintain eggs in suspension by the water turbulence. However, these suspension velocities were obtained by observation of known spawning grounds, and the critical stage for Asian carp recruitment is when the eggs are water-hardened. More recent studies show a better approximation to the critical velocity to maintain eggs in suspension. Murphy and Jackson [12] determined through a hydraulic-based analysis of shear velocity versus settling velocity, that eggs will start to sink at mean velocities on the order of 0.[15]^m^/_s_ to 0.[25]^m^/_s_. Using the FluEgg drift model, Garcia et al. [13] showed substantial settling of water hardened silver carp eggs when the mean velocity fell below 0.[16]^m^/_s_. Further downstream, at velocities approaching 0.03^m^/_s_, the vertical centroid of the mass of eggs was approximately in the lower 10% of the water column. The results of the present laboratory study indicate that for the conditions evaluated in the experiments, water-hardened silver carp eggs will remain in suspension at velocities as low as 0.07^m^/_s_ (u* = 0.004^m^/_s_). This is an order of magnitude lower than critical suspension velocities used in Kocovsky et al. [11]. However, generalization and identification of a single critical velocity to maintain eggs in suspension is difficult because maintaining eggs in suspension depends on the amount of turbulent fluctuations present throughout the flow. These turbulent fluctuations are, in turn, dependent on flow velocity, water depth, bed morphology, bed substrate, and water temperature, among other factors. Consequently, the critical velocity for maintaining eggs in suspension at a specific developmental stage may vary depending on the local bathymetry and water temperature. However, results from this laboratory experiment suggest that water-hardened silver carp eggs will likely remain in suspension at relatively low velocities. These observations help delineate the bounds on the magnitude of the critical velocity necessary to maintain water-hardened eggs in suspension.

### Drift trajectories of synthetic surrogate eggs

One important question regarding suspension and settling dynamics of eggs, is whether an egg will remain in its initial vertical location in the water column for a long period of time under steady state conditions. To answer this question it might be useful for biologists and scientists to determine whether eggs collected near the surface have been drifting for an extended period of time, or had periods of settling and resuspension upstream of a sampling point. The former might allow estimation of spawning sites from velocity data and egg development stage, while the latter would introduce added complexity. Analyzing the drift trajectories of synthetic eggs in the laboratory is not only useful to answer questions similar to the one above, but also to give insights regarding why an egg driven by the turbulence and mixing processes will tend to go upwards rather than settling down due to gravity. The trajectory slopes provide information regarding the balance between settling forces and upward turbulent and mixing forces.

This set of experiments were performed in the flume under relatively flat bed conditions and velocities equal to 0.07^m^/_s_ (u* = 0.004^m^/_s_). Egg trajectories were derived by tracking correlated frames taken at a rate of 8 frames per second. The location of the centroid of every egg in every picture frame was detected using the PTVlab toolbox. Then, particles were tracked between pairs of pictures. This tracking was performed by computing the cross-correlation coefficient of light intensities between a pair of picture frames, and using an interrogation area of about 3 times the diameter of the synthetic eggs.

**Fig 7:**
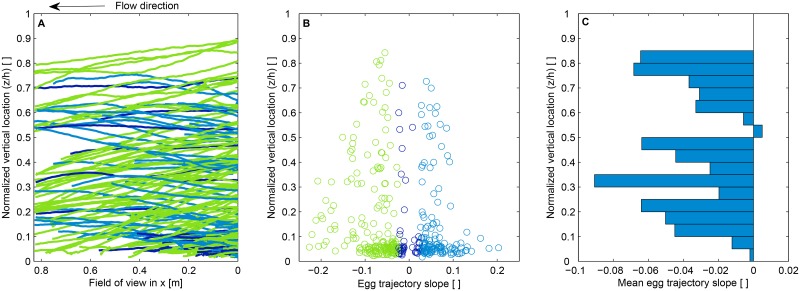
Synthetic egg trajectories using correlated images taken at 8 frames per second (A). Slope of trajectories as a function of normalized egg mean location (B). In (A) and (B), colors indicate negative (green), positive (light blue), and mild slope (dark blue) of the egg trajectories. Mean egg trajectory slopes as a function of normalized egg mean location (C).

Trajectories of synthetic eggs are illustrated in Fig 7A. Trajectories were categorized by their mean slope: negative (green), positive (light blue), and mild slope (dark blue). Negative slopes indicate the case where mixing is not sufficient to counteract settling effects. Mild slopes indicate that there is a general balance between turbulent mixing and settling forces; therefore, the vertical locations of the synthetic eggs remain relatively stable across the field of view of the camera. Positive slopes indicate that turbulent mixing overcomes settling processes; therefore, synthetic eggs move upward in the water column. In Fig 7B, the mean slope of egg trajectories are presented as a function of the normalized egg mean vertical location. The distribution of the mean slope of synthetic egg trajectories over the normalized vertical location of the eggs is shown in Fig 7C. The vertical distribution of the mean trajectory slopes illustrate the combined effect of velocity gradients and turbulent mixing (eddy diffusivity). The overall tendency of the majority of synthetic eggs is to go downward, which indicates that at a velocity of 0.07^m^/_s_ (u* = 0.004^m^/_s_) and under relatively flat bed conditions, the turbulent mixing is not very strong and the eggs are settling in the test section. This is consistent with the finding that u*<*V*_*s*_. However, there is a zone in Fig 7C that indicates turbulent mixing is stronger. This zone corresponds to the layer that is located at about 55% (z/h = 0.55) of the water column depth. In this layer there are still synthetic eggs that tend to settle; however, there is a slightly higher tendency of synthetic eggs to move upward. This weak upward tendency suggests that the turbulent fluctuations in this portion of the water column have just overcome the settling effects of the mechanism that drives the trajectories of the eggs. The layer closest to the bed illustrates an overall stable tendency of the location of the eggs because relatively equal amounts of eggs tend to rise and settle (possibly due to saltation). Near the bed, turbulent mixing is low compared to the rest of the water column; therefore, eggs will tend to remain in this area for longer periods of time.

### Analysis of suspension of synthetic surrogate eggs with dimensionless numbers

Estimates of shear velocity in the present experiments were used to further analyze the relation between flow condition and egg suspension. For this task, the shear velocities and the vertical turbulence intensity ($\tilde{w}$) were used to calculate three dimensionless ratios. The turbulence intensity is expressed as [31]:

$$\begin{array}{c} \tilde{w}={\left[\bar{{\left(w^{\prime }\right)}^{2}}\right]}^{0.5} \end{array}$$ (3)

where *w*′ is the fluctuation of the vertical velocity component [^m^/_s_].

The dimensionless numbers selected to analyze the autosuspension thresholds (the turbulence limit at which eggs are held in suspension) were i) the ratio between the vertical turbulence intensity and the settling velocity $\left(\frac{\tilde{w}}{V_{s}}\right)$, ii) the ratio between the shear velocity and settling velocity $\left(\frac{u*}{V_{s}}\right)$, and iii) the inverse of the Rouse number $\left(\frac{1}{Z_{R}}\right)$.

Van Rijn [31] defined the first two dimensionless numbers $\left(\frac{\tilde{w}}{V_{s}}\;\text{and}\;\frac{u*}{V_{s}}\right)$ as parameters that indicate initiation of suspension when greater or equal to one (binary suspension screening criteria). Similarly, in a previous study performed by Murphy and Jackson [12], the shear velocity was compared against the settling velocity of water-hardened Asian carp eggs to identify potential settling zones of Asian carp eggs in Great Lakes tributaries. Although the authors did not calculate directly $\frac{u*}{V_{s}}$, they used $\frac{u*}{V_{s}}<1$ as a criteria to identify potential settling zones.

In sediment transport the Rouse number is used to determine the mode of transport (e.g., bedload, suspended load, wash load) and its inverse $\left(\frac{1}{Z_{R}}\right)$ is call the suspension parameter [32]. The inverse of the Rouse number is defined as follows [31]:

$$\begin{array}{c} \frac{1}{Z_{R}}=\frac{\beta \kappa u_{*}}{V_{s}} \end{array}$$ (4)

where *κ* = 0.51 is the Von Karman constant [*unitless*] and $\beta =1+2{\left(\frac{V_{s}}{u_{*}}\right)}^{2}$ is a coefficient related to diffusion of sediment particles [*unitless*] with a maximum value of 3.

Hearn [32] classifies sediment transport modes as follows: bed load $\left(\frac{1}{Z_{R}}<0.4\right)$, suspended load with approximately 50% of sediments in suspension $\left(0.4\leq \frac{1}{Z_{R}}\leq 0.8\right)$, suspended load with approximately 100% of sediments in suspension $\left(0.8<\frac{1}{Z_{R}}\leq 1.3\right)$, and wash load (*Z*_*R*_>1.3). The classification of transport mode proposed by Hearn [32] was used to compare synthetic egg transport modes in experiments performed in this study.

**Fig 8:**
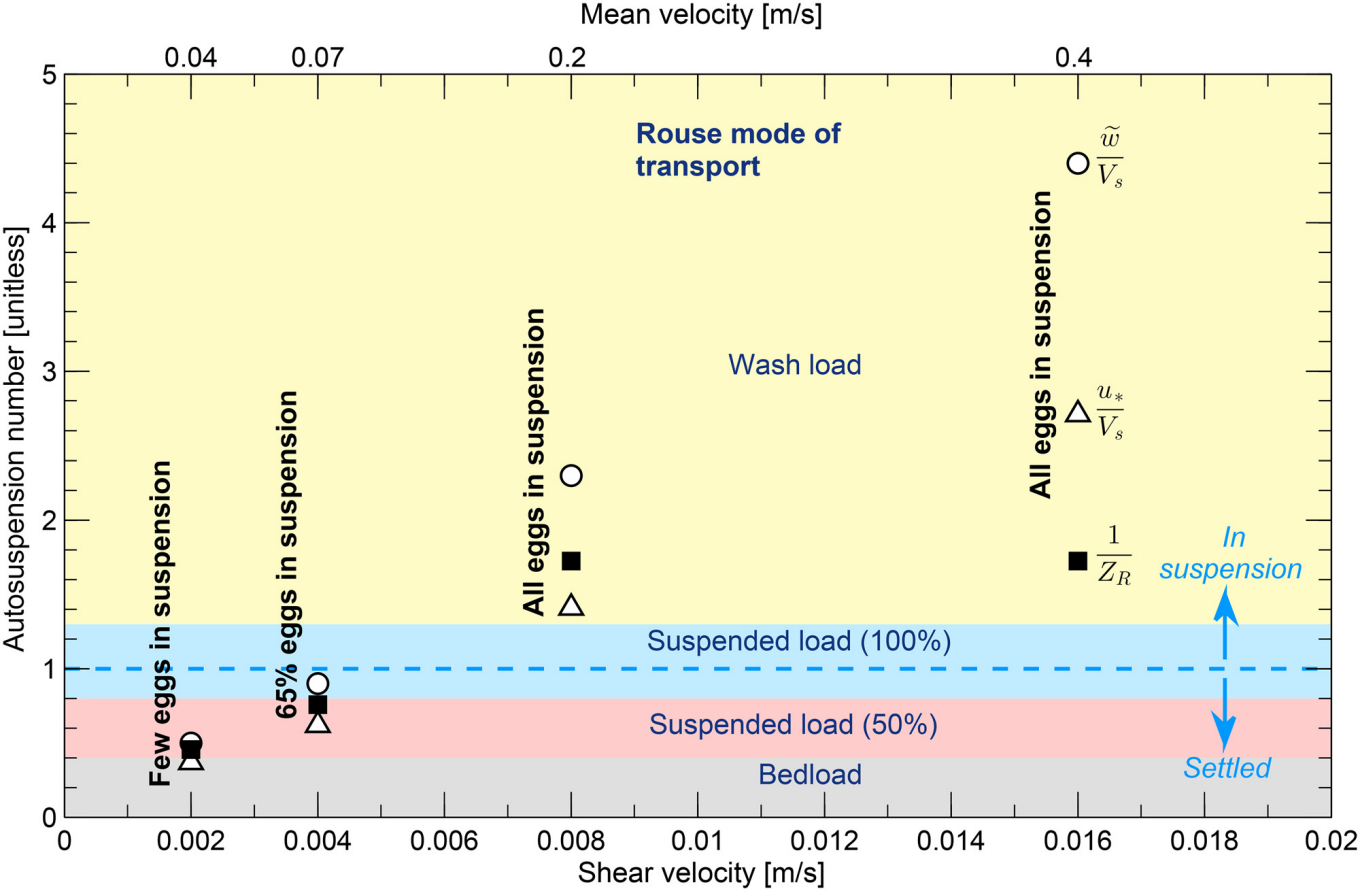
Autosuspension dimensionless numbers calculated for synthetic eggs drifting at different flow conditions. Qualitative and quantitative results from experiments with synthetic eggs are indicated with black labels. Circles represent $\frac{\tilde{w}}{V_{s}}$, triangles represent $\frac{u*}{V_{s}}$, and black squares represent $\frac{1}{Z_{R}}$ for the different flow conditions: 0.04^m^/_s_ (u* = 0.002^m^/_s_), 0.07^m^/_s_ (u* = 0.004^m^/_s_), 0.2^m^/_s_ (u* = 0.008^m^/_s_), and 0.4^m^/_s_ (u* = 0.016^m^/_s_). Light dashed blue line and arrows represent the binary autosuspension criteria applicable to $\frac{\tilde{w}}{V_{s}}$ and $\frac{u*}{V_{s}}$. Shaded colored areas represent the different Rouse mode of transport conditions: bedload(gray), suspended load (50%) (pink), suspended load (100%) (blue), wash load (yellow).

The calculated autosuspension numbers ($\frac{\tilde{w}}{V_{s}}$,$\frac{u*}{V_{s}}$, and $\frac{1}{Z_{R}}$) for the synthetic eggs at different flow conditions are illustrated in Fig 8 together with the binary suspension screening criteria and the Rouse classification of mode of transport for comparison purposes. Autosuspension numbers based on u* are likely to be sensitive to the method used for estimating u*. The first two autosuspension dimensionless numbers were compared against the binary suspension screening criteria proposed by Van Rijn [31] (dashed blue line in Fig 8), in which either the turbulence intensity or the shear velocity are compared against the settling velocity of synthetic eggs, therefore explaining the correlation between egg suspension and the turbulence of the flow. Results show that these two autosuspension numbers successfully characterize the transport mode where all the eggs are in suspension. However, these dimensionless numbers failed to describe the condition where some eggs remain in suspension, yet $\frac{u*}{V_{s}}<1$. The experiments with synthetic eggs depicted some suspension of the eggs (up to 65%) when u*<*V*_*s*_. The binary suspension screening criteria are useful for estimating when eggs start to fall out of suspension, but can not adequately describe the amount of eggs that remain in suspension after some of the eggs start to settle.

The suspension parameter adequately described the mode of transport of synthetic eggs for the different flow conditions in experiments with moving water (see Fig 8). For the case of mean velocity equal to 0.04^m^/_s_ (u* = 0.002^m^/_s_), the suspension parameter was equal to 0.45, which is near the limit of bedload transport, consistent with just a few synthetic eggs in suspension. For the case of mean velocity 0.07^m^/_s_ (u* = 0.004^m^/_s_), at which 65% of eggs were in suspension, the suspension parameter was equal to 0.76, which is within the suspended load (50% suspended) classification limits. Finally, for the last two flow conditions (mean velocity equal to 0.2^m^/_s_ (u* = 0.008^m^/_s_) and 0.4^m^/_s_ (u* = 0.016^m^/_s_)) the suspension parameter corresponds to 1.74 and 1.72, respectively, which predicts 100% suspension and agrees with the experiments with synthetic eggs in which all the eggs were in suspension. Therefore using the suspension parameter, or inverse of the Rouse number, might be appropriate to describe egg transport mode, especially within flows where settling is possible.

## Conclusions

Under the assumption that Asian carp will spawn in highly turbulent areas, with enough mixing to keep the non-water hardened eggs in suspension, we identified water-hardened silver carp eggs as the critical indicator to analyze the hydrodynamic conditions necessary to maintain Asian carp eggs in suspension long enough to facilitate egg development and hatching. In previous studies, scientists employed a velocity threshold based on known spawning grounds to evaluate tributaries suitable for egg development and incubation. This practice resulted in a high velocity threshold which does not accurately represent the minimum velocities required to maintain water-hardened eggs in suspension. Ultimately, this practice can lead to incorrect conclusions and decisions regarding the suitability of a stream for development and hatching of Asian carp eggs.

Styrene particles in salt water with a mean diameter of 4.85mm (representative of water-hardened silver carp eggs) and a density and specific gravity of about 1040.9^kg^/_m^3^_ and 1.043, respectively, were found to have a similar fall velocity to water-hardened silver carp eggs. The salinity of the salt water was equal to 55.5 ppt with a specific gravity equal to 1.042. The terminal settling velocity of synthetic eggs was calculated experimentally with a vertical settling column using an image processing technique. The terminal settling velocity of the synthetic eggs was equal to 0.006±0.0016^m^/_s_; very close to that of water-hardened silver carp eggs (0.007±0.0006^m^/_s_, [21]). With small adjustments in the salinity of the water and particle size, this technique should allow one to mimic eggs at different developmental stages, eggs of different species, or other passive particles (e.g., sediment grains and oil droplets).

Qualitative observations of synthetic eggs drifting at a range of velocities and under different bed configurations generated insights regarding egg suspension, saltation processes, and interaction of the eggs with the bed. At velocities lower than 0.07^m^/_s_ (u* = 0.004^m^/_s_), synthetic eggs were trapped in the recirculation zone on the leeward side of bedforms and near the walls of the flume where the velocities were lower. Saltation or bouncing of the eggs along the bed was observed at velocities greater than or equal to 0.07^m^/_s_ (u* = 0.004^m^/_s_). At lower velocities, saltation processes were limited to rolling of the non-trapped eggs on top of the sediment bed. Finally, at velocities higher than 0.2^m^/_s_ (u* = 0.008^m^/_s_), differences between the bedforms and flat bed conditions were not noticeable.

One of the main conclusions of this study is that at mean and shear velocities as low as 0.07^m^/_s_ and 0.004^m^/_s_, respectively, over a relatively flat sediment bed, about 65% of synthetic eggs remain in suspension. This mean velocity is about 10 times lower than the velocity required for suspension previously cited in literature based on known spawning grounds. Factors including temperature, bed roughness, bed substrate, and presence of bedforms will also affect egg suspension behavior and must be considered, in addition to the mean and shear velocity.

Results from qualitative and quantitative experiments with synthetic eggs were compared against three different dimensionless autosuspension numbers ($\frac{\tilde{w}}{V_{s}}$,$\frac{u*}{V_{s}}$, and $\frac{1}{Z_{R}}$) with the goal of identifying a screening method that adequately predicts egg transport (suspension or settling) to be used for first order analysis of rivers for reproduction suitability. The three assessed autosuspension numbers appropriately described the cases where all the eggs were in suspension. However, $\frac{\tilde{w}}{V_{s}}$ and $\frac{u*}{V_{s}}$ predicted settling of all the eggs for flow conditions where suspension of some eggs was observed (e.g., at velocities equal to 0.07^m^/_s_), therefore these autosuspension numbers should only be used to identify areas of transport (suspension) and areas where eggs may settle (u*<*V*_*s*_, settling zone). Because rivers and streams have streamwise gradients in velocity and turbulence intensity and, therefore, do not adhere to strict binary autosuspension criteria, it is important to note that once a potential settling zone is identified, use of the suspension parameter is advised. In reality, eggs will not settle in unison upon reaching a settling zone and this behavior is captured by transport modes of the suspension parameter. Finally, results from these laboratory experiments demonstrate the delicate balance between the combined effects of velocity gradients and turbulent mixing and particle settling in the suspension of synthetic Asian carp eggs. Although the applications of the study to riverine environments are limited by the experimental setup (particularly, the narrow flume), the knowledge gained from this study is useful to help delineate the boundaries surrounding the critical hydrodynamic conditions of the flow at which water-hardened silver carp eggs fall out of suspension.

Future work is needed on the transport dynamics at or near the threshold for suspension, particularly with regard to the applicability of the suspension parameter and Rouse number (originally developed for sediment transport) to biological particles such as eggs. The present results suggest the suspension parameter may provide a more accurate assessment of egg transport compared to binary autosuspension parameters or a mean velocity threshold. Use of the suspension parameter may lead to more accurate assessments of potential spawning rivers. Laboratory experiments should be extended to improve the knowledge on the transport and dispersal patterns of Asian carp eggs in flowing water for a range of different conditions including egg development, water temperature, sediment type, bed structure, and carp species. In addition, experiments with fertilized Asian carp eggs should be considered.

## Supporting Information

S1 Video Video of synthetic eggs drifting under different flow conditions and bed configurations. (MP4)
